# LncRNA AC099850.3 promotes hepatocellular carcinoma proliferation and invasion through PRR11/PI3K/AKT axis and is associated with patients prognosis

**DOI:** 10.7150/jca.66092

**Published:** 2022-01-04

**Authors:** Fangjing Zhong, Sheng Liu, Donghai Hu, Liwen Chen

**Affiliations:** 1 Department of Anesthesiology, Hubei Cancer Hospital, Wuhan 430079, China; 2 Department of Hepatobiliary Surgery, Hubei Cancer Hospital, Wuhan 430079, China; 3 Department of Hepatopancreatobiliary Surgery, The Third Xiangya Hospital, Central South University, Changsha 410000, Hunan, China

**Keywords:** LncRNA, AC099850.3, hepatocellular carcinoma, PRR11, PI3K/AKT, tumor immunity

## Abstract

**Background:** LncRNA is a key factor influencing tumor development. The present study aimed to investigate the effect of a novel lncRNA on the progression of hepatocellular carcinoma (HCC).

**Methods:** A candidate lncRNA in The Cancer Genome Atlas database was identified using limma and survival R packages. The effect of lncRNA AC099850.3 on cell proliferation, apoptosis, migration, and invasion, as well as its association with immune cells in HCC were investigated. Furthermore, the functional mechanisms of lncRNA AC099850.3 in HCC were elucidated.

**Results:** The aberrant expression of lncRNA AC099850.3 was identified in tumor tissues and its prognostic relevance in HCC was determined. The results revealed that AC099850.3 was highly expressed in HCC tissues and cell lines, and it predicted poor prognosis in patients with HCC. Furthermore, knockdown of AC099850.3 significantly suppressed the proliferation and metastatic potential of HCC cells, and promoted cell apoptosis in HCC cells. The results of gene set enrichment analysis revealed that the PI3K/AKT pathway was associated with the biological function of AC099850.3, which was further validated by western blotting. *PRR11* was identified as the target gene of AC099850.3 and we established that AC099850.3 acted as an oncogene in the PRR11/PI3K/AKT axis. Immune cell infiltration analyses results revealed that AC099850.3 was positively correlated with T follicular helper cells, M0 macrophages, CD4^+^ memory T cells, and memory B cells. Conversely, AC099850.3 was negatively correlated with M2 macrophages, monocytes, natural killer cells, and CD8^+^ T cells, which could be responsible for its oncogenic effect. Of note, a significantly positive correlation was observed between AC099850.3 and key immune checkpoint molecules (PD-1, PD-L1, PD-L2, and CTLA4) in the present study, making AC099850.3 a potential immune therapeutic target for HCC.

**Conclusion:** AC099850.3 can promote malignant biological behavior of HCC cells, and could be a potential biomarker and therapeutic target for HCC.

## Introduction

Liver cancer is one of the most prevalent malignant tumors and the third leading cause of cancer-related death worldwide. The ten-year survival rate of liver cancer is approximately 20% 1. Based on the pathological subtypes, hepatocellular carcinoma (HCC) is the most widespread subtype of liver cancer and accounts for approximately 90% cases 2. Chronic hepatitis remains the leading cause of HCC and most patients with HCC are often diagnosed at advanced stages due to lack of early symptoms 3. Hepatectomy is an effective approach for the treatment of patients with HCC. However, high rates of postoperative metastasis and recurrence, as well as liver function reserve of patients with advanced stage HCC are still some of the clinical challenges associated with poor prognosis 4, 5, which highlights the importance of developing a novel therapeutic strategy. Currently, molecular targeted therapy has shown a promising prospect in tumor therapy and prognosis improvement.

Long non-coding RNAs (LncRNAs) are a type of non-coding RNAs with lengths exceeding 200 nucleotides and they can influence the maturity of human cells, metabolic states, and physical conditions 6. LncRNAs primarily serve as regulators in human cells and function by regulating the downstream genes and proteins 7. In addition, increasing attention has been focused on the underlying association between lncRNAs and malignant tumors. For example, Lian et al. identified a novel lncRNA LINC00460 in colorectal cancer, which facilitated cell proliferation and inhibited apoptosis by regulating KLF2 and CUL4A 8. Li et al. demonstrated that the lncRNA SNHG1 could influence sorafenib resistance by activating the AKT pathway, which had the potential for clinical application 9. Zhou et al. revealed that the LncRNA ID2-AS1 could inhibit the migration, invasion, and metastasis of HCC through the HDAC8/ID2 pathway based on *in vitro* and *in vivo* experiments 10.

Presently, the rapid development of next-generation sequencing generates massive genomic data, which facilitates the analysis of intrinsic disease mechanisms. In the current study, we first identified a prognosis-related lncRNA AC099850.3 by performing bioinformatics analysis. *In vitro* and* in vivo* experiments were further conducted to elucidate the role of AC099850.3 in HCC. The results revealed that AC099850.3 was highly expressed in HCC tissues and cell lines, and it was associated with poor prognosis in patients with HCC. AC099850.3 significantly promoted HCC cell proliferation, migration, and invasion through the PRR11/PI3K/AKT pathway. Moreover, correlation analyses results revealed that AC099850.3 considerably influenced the tumor immune microenvironment of HCC and it was positively associated with the expression of immune checkpoints molecules (PD-1, PD-L1, PD-L2, and CTLA4). Immunofluorescence analysis revealed that tissues with high expression of AC099850.3 tended to have relatively low M2 macrophage infiltration levels. In summary, our results revealed that AC099850.3 could be a potential therapeutic target for HCC.

## Methods

### Data acquisition

The open access data used in the present study, including transcriptome data, clinical information, and information regarding prognosis of patients with hepatocellular carcinoma (TCGA-LIHC) were obtained from The Cancer Genome Atlas (TCGA, https://portal.gdc.cancer.gov/). Transcriptome data in the form of “FPKM” (Fragments per kilobase of exon model per million mapped reads) and annotated by the last reference file “GRCh38.p13,gct” were downloaded from the ENSEMBL website (http://useast.ensembl.org/index.html). The expression profiles were processed by completing missing values and normalizing data before analysis. Clinical and prognostic information of single samples were collated into a combined file using R software version 3.6.0 (The R Foundation for Statistical Computing, Vienna, Austria/ https://www.r-project.org). The patients of TCGA meeting the following criteria were included in our analysis: 1. have transcriptional profiling data; 2. have complete clinical information. The patients with incomplete clinical information and transcriptional data were excluded.

### Identification of differentially expressed lncRNAs and clinical correlation analysis

Genes included in the expression profiles were separated into protein-coding and lncRNAs genes based on the gene-biotype in the reference file “GRCh38.p13,gct”. Differentially expressed lncRNAs (DELs) were identified using limma package in R with a threshold value of |logFC|>3 and P value < 0.05. Wilcoxon test for paired samples was used to compare the differences in target lncRNAs between two groups. Survival package in R was used to analyze differences in prognosis and Kaplan-Meier survival curves were used to analyze differences between two groups.

### Tissues and cell lines

HCC and adjacent normal tissue were obtained from Hubei Cancer Hospital, which were pathological confirmed. All patients provided written informed consent. Four HCC cell lines (HepG2, Hep3B, Huh7, and LM3) and one normal human hepatocyte cell line (LO2) were purchased from iCell Bioscience Inc. (Shanghai, China).

### Quantitative real-time polymerase chain reaction assay

Total RNA of HCC tissues and cell lines were extracted using RNA extraction kit (Tiangen Biotech., Beijing, China) according to the manufacturer's instructions. Subsequently, reverse transcription was performed using FastQuant RT kit (Tiangen Biotech., Beijing, China). Polymerase chain reaction (PCR) amplicons were detected using SYBR green real-time quantitative PCR (qRT-PCR). The primers used were as follows: AC099850.3-F: 5'-CCCAGGTTCAAGTAACTGGGAC-3'; AC099850.3-R: 5'-GACAATGCCTTGCCAAGGAATC-3'; CD274-F: 5'-CTGCCTTGTTCCTGAGAGTGAAG-3'; CD274-R: 5'-CATCGTACTCCTCTCTTCGTCC-3'; PDCD1-F: 5'-AAGGCGCAGATCAAAGAGAGCC-3'; PDCD1-R: 5'-CAACCACCAGGGTTTGGAACTG-3'; CTLA4-F: 5'-CATGATGGGGAATGAGTTGACC-3'; CTLA-R: 5'-TCAGTCCTTGGATAGTGAGGTTC-3'; PDCD1LG2-F: 5'-CTCGTTCCACATACCTCAAGTCC-3'; PDCD1LG2-R: 5'-CTGGAACCTTTAGGATGTGAGTG-3'; GAPDH-F: 5'-CATGGGTGTGAACCATGAGA-3'; GAPDH-R: 5'-CAGTGATGGCATGGACTGTG-3'.

### Western blotting assay

Total proteins were extracted using the total protein extraction kit (P1250; Applygen Technologies Inc., Beijing, China) and their concentrations were determined using a BCA protein assay kit (Beyotime Biotechnology, Shanghai, China). Protein samples were initially separated using dodecyl-sulfate polyacrylamide gel electrophoresis and then transferred onto polyvinylidene fluoride membranes. The polyvinylidene fluoride membranes were incubated with primary antibodies (anti-AKT, anti-p-AKT, anti-cyclin D1, and anti-GAPDH) overnight at 4 °C. Afterward, the membranes were incubated with secondary antibodies for 30 min at room temperature.

### Cell transfection assay

Cells were transfected with Lipofectamine 2000 transfection reagent that was purchased from Invitrogen Corp (Carlsbad, CA, USA). The control and target plasmids were transfected into Hep3B and Huh7 cell lines. The target sequences of siRNAs were follows: AC099850.3-siRNA1: CTGCTATGGACTTCAGAGA; AC099850.3-siRNA2: CCAGGCTGTATTACTGTCT; AC099850.3-siRNA3: GCGTCACCATGCCTGGGTA. The knockdown efficiencies were verified using qRT-PCR.

### Colony formation assay

Colony formation assay was performed using Hep3B and Huh7 cell lines. A total of 500 cells were seeded into a six-well plate and cultured for two weeks. Culture medium was changed every four days. Cells were finally stained with crystal violet and counted.

### Cell counting kit-8 assay

Cell proliferation/viability assay was conducted using a cell counting kit-8 (CCK-8 kit) (Dojindo, Kumamoto, Japan). Briefly, cells were resuspended in serum-free medium and then inoculated into 96-well plates at a density of 200 cells per plate. Plates were incubated at 37 °C for 2 hours and absorbance measured at a wavelength of 450 nm.

### EdU (5-Ethynyl-2-deoxyuridine) assay

EdU assay was conducted to assess DNA synthesis in cells using the EdU assay kit (RiboBio, Guangzhou, China). Briefly, cells were incubated with EdU solution for 2 hours under the condition of 5% CO_2_ and 37 °C. Thereafter, cells were fixed with 4% paraformaldehyde and protected from light. After staining with DAPI, cells were observed and photographed by fluorescence microscopy.

### Analysis of cell apoptosis using flow cytometry

Cell apoptosis was assessed using an Annexin V-FITC/PI apoptosis kit (KeyGen Biotech, Nanjing, China). Cells were digested with trypsin without EDTA and washed three times with phosphate-buffered saline. Cells were resuspended in binding buffer and then stained with anti-Annexin V-FITC/PI. A flow cytometer was used to analyze the rate of apoptosis in cells.

### Transwell migration and invasions assays

Transwell invasion and migration assays were performed in 24-well chambers (Corning Costar, Cambridge, MA, USA). Cells were resuspended in serum-free medium and then placed in the upper chamber with or without Matrigel. Medium (500 ul) containing 10% serum was added to the lower chambers. The invaded cells were stained with crystal violet after 24 hours.

### Wound healing assay

Cells were seeded into six-well plates and cultured to a confluent monolayer. The confluent monolayer of cells was scratched using a 10-µl pipette tip. Cell scratches were recorded using a microscope camera at 0 and 24 hours.

### Immunofluorescence

Immunofluorescence was used to assess the level of M2 macrophage infiltration in patients with high and low AC099850.3 expression. Tissue sections were fixed in 4% paraformaldehyde and then blocked with 2.5% goat serum at room temperature for 1 hour. Next, the sections were incubated with CD206 antibody (proteintech) overnight at 4 °C. After that, the sections were incubated with second antibody (anti-rabbit IgG) for 1 hour and with DAPI for 15 min at room temperature. A confocal microscope was used to perform slices photograph.

### Biological pathway and immune infiltration analyses

Gene set enrichment analysis (GSEA) was used to identify the biological pathways associated with AC099580.3. The reference gene set was “Hallmark.v7.4.gmt”. CIBERSORT algorithms were used to quantify 22 immune cells in tumor tissues. The CIBERSORT reference and data source used in this study have been uploaded in Figshare (https://figshare.com/articles/dataset/CIBERSORT/16908028). Spearman's correlation test was used to assess the correlation between AC099850.3 and immune cells. The correlation between AC099850.3 and three immune checkpoints was analyzed through the GEPIA database (http://gepia.cancer-pku.cn/index.html).

### *In vivo* xenograft experiments

The protocols for animal experiments were approved by the Animal Ethics Committee of Central South University, Changsha, China. Briefly, 5 × 10^6^ Huh7 cancer cells were implanted into BALB/c nude mice (n = 5 per group). All the mice received a standard laboratory diet for four weeks. Afterward, the mice were euthanized and tumor tissues were harvested. The tumor sizes were calculated using the following formula: Tumor volume (mm^3^) = (length × width^2^)/2.

### Immunostaining (IHC) and HE staining

Xenograft tumors were firstly fixed in 4% paraformaldehyde and then dehydrated with ethanol. Next, tumor specimens were cut into sections with 5 um thick. The tissue sections were then subjected to the following treatments: 1. were mounted on glass microscope slides; 2. deparaffinized in xylene; 3. rehydrated in a graded alcohol series; 4. antigen repair in high temperature. Furthermore, the sections were cooled, rinsed, and endogenous peroxidases were quenched using 3% H_2_O_2_. Then, the sections were incubated with 5% BSA for 40min at room temperature. After that, the sections were incubated in the KI67 antibody (1:2000; Proteintech) overnight at 4 °C. Next, the sections were washed and incubated in secondary antibody for 1h at room temperature, whose staining was then visualized through EnVision system (EnVision; Dako, Glostrup, Denmark). Two pathologists independently and blindly interpreted the IHC using a semi-quantitative integration method. Detailed, the proportion of KI67 positive cells was scored as 1 = 0 ~ 10%, 2 = 10% ~ 25%, 3 = 50% ~ 75%, and 4 = 75% ~ 100%. Staining intensity was scored as 0 = no staining, 1 = weak staining, 2 = moderate staining, and 3 = strong staining. The final IHC score was a product of two scores. Score larger than six were regarded as a high score and low when equal to or less than six. HE staining was performed using a HE Staining kit (Beyotime, China).

### Statistical analysis

Statistical analyses were conducted using IBM SPSS Statistics for Windows v22.0 (IBM Corp., Armonk, NY, USA), GraphPad Prism 7 (GraphPad Software Inc., La Jolla, CA, USA) and R software (The R Foundation for Statistical Computing, Vienna, Austria/ https://www.r-project.org). All experiments were repeated at least three times. Tests for statistical significance were two-sided, and P value ≤ 0.05 was considered significant.

## Results

### Identification of candidate lncRNAs in HCC cells

**Figure 1:**
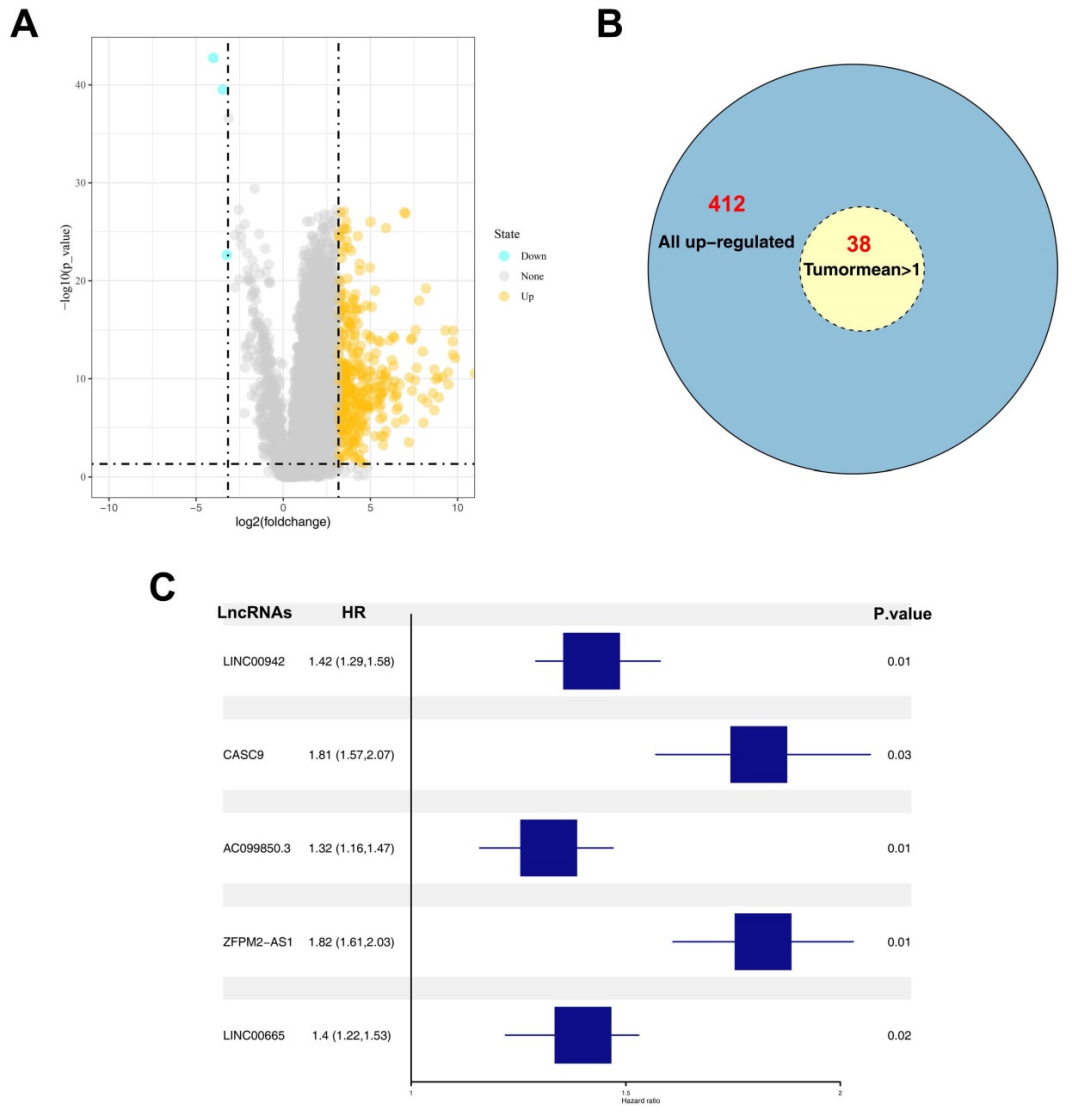
Identification of AC099850.3 as a candidate gene. **Notes: A:** The volcano plot of TCGA-LIHC between normal and tumor group; **B:** Overview of 412 up-regulated lncRNAs; **C:** Five prognosis-related lncRNAs identified by multivariate cox analyses. **Abbreviations:** TCGA, The Cancer Genome Atlas

**Figure 2:**
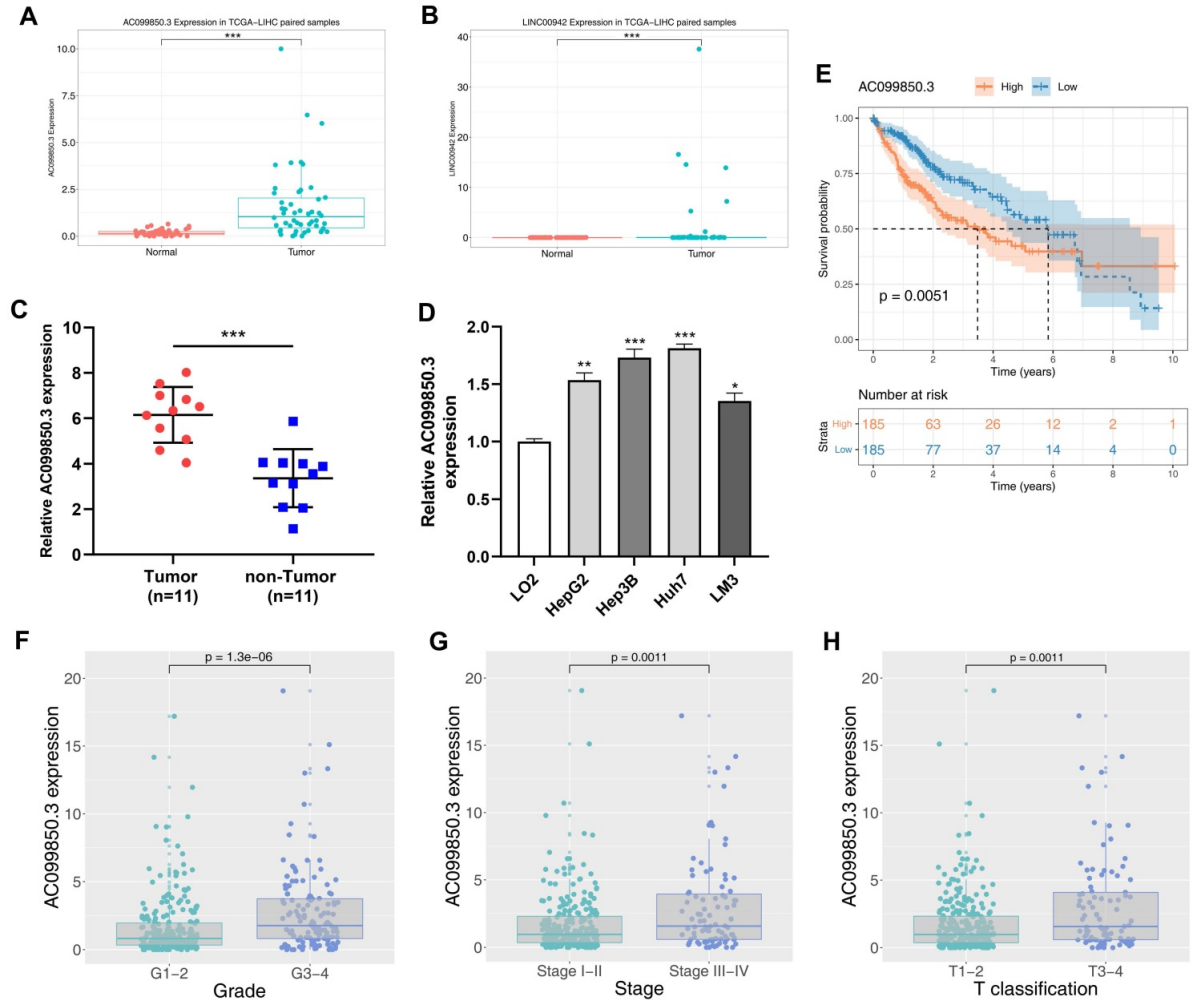
Clinical correlation and prognosis analysis of AC099850.3. **Notes: A:** The expression of AC099850.3 in TCGA-LIHC; **B:** The expression of LINC00942 in TCGA-LIHC; **C:** The expression pattern of AC099850.3 in 11 paired HCC tissue relative to GAPDH; **D:** The expression of AC099850.3 in normal and HCC cell lines relative to GAPDH; **E:** Kaplan-Meier curve of OS in high and low AC099850.3 group; **F-H**: Clinical correlation of AC099850.3. **Abbreviations:** OS, Overall survival; TCGA, The Cancer Genome Atlas.

A total of 412 up-regulated and 4 down-regulated lncRNAs were identified based on a threshold value of |logFC|>3 and P value < 0.05 (***Figure 1A***). Considering the bias caused by extremely low expression values, DELs whose average values in tumor samples were > 0.1 were selected for further analysis. Among the 412 up-regulated lncRNAs, 38 lncRNAs satisfied the selection criteria (***Figure 1B***). Univariate and multivariate analyses were performed to identify prognosis-related DELs, and five DELs (LINC00942, CASC9, AC099850.3, ZFPM2-AS1, and LINC00665) were finally identified (***Figure 1C***). Among the five DELs, CASC9, ZFPM2-AS1, and LINC00665 have been reported to influence the biological behavior of liver cancer cells 11-13. Furthermore, relatively high expression levels of AC099850.3 and LINC00665 were observed in tumor samples (***Figure 2A-B***). However, most tumor samples in TCGA-LIHC hardly expressed LINC00665, and its high expression in tumor tissues was probably due to several samples having extremely high values, which could lead to a significant bias in its correlation with prognosis. Therefore, lncRNA AC099850.3 was selected for subsequent analysis.

### AC099850.3 is highly expressed in HCC cells and it is correlated with worse clinical features

The clinical features of enrolled patients were shown in ***Table 1***. We examined the expression of AC099850.3 in eleven paired HCC samples and four HCC cell lines to verify its expression in HCC cells. Relatively high expression levels of AC099850.3 were observed in HCC tissues and cell lines based on TCGA data (***Figure 2C-D***). In addition, the results revealed that patients with high expression levels of AC099850.3 tended to have a worse overall survival based on clinical information obtained from TCGA (***Figure 2E***; P = 0.0051). A high expression level of AC099850.3 was associated with worse clinical features, including pathological grade, clinical stage, and T classification (***Figure 2F-H***).

### Knockdown of AC099850.3 inhibits the proliferation of HCC cells

**Figure 3:**
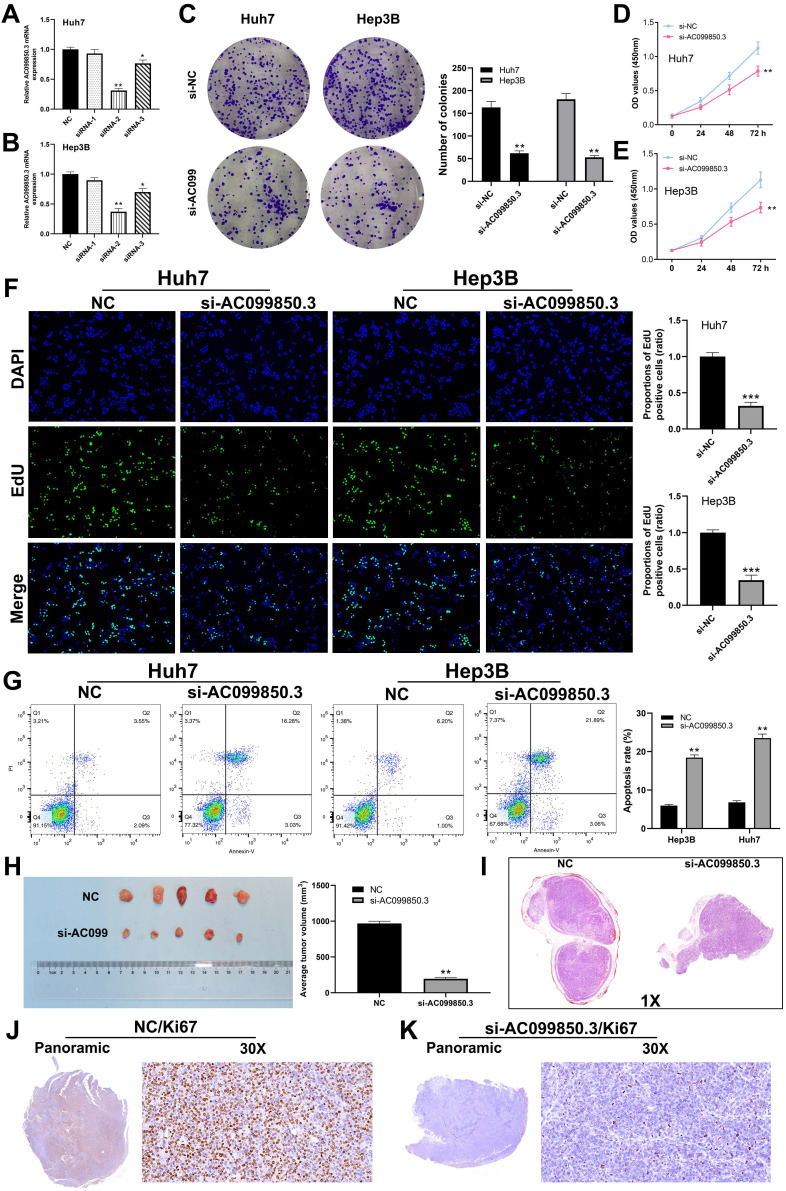
AC099850.3 promotes cell proliferation in HCC. **Abbreviations: A-B:** The knockdown efficiency of AC099850.3 validated by qRT-PCR relative to GAPDH; **C:** Colony information assay performed in NC and si-AC099850.3 group; **D-E:** CCK8 assay performed in NC and si-AC099850.3 group; **F:** Edu assay performed in NC and si-AC099850.3 group; **G:** Flow cytometry detected cell apoptosis in NC and si-AC099850.3 group;** H:** In vivo experiment of NC and si-AC099850.3 group; **I:** HE staining of the xenograft tumors; **J-K:** Ki67 staining of the xenograft tumors; **Notes:** All experiments were repeated at least three times

To determine the role of AC099850.3 in HCC, Hep3B and Huh7 cell lines were used to knock down AC099850.3 for their highest AC099850.3 expression level. qRT-PCR revealed that AC099850.3-siRNA2 exhibited the highest knockdown efficiency and was selected for subsequent experiments (***Figure 3A-B***). Colony formation assay revealed that knockdown of AC099850.3 significantly suppressed the proliferation ability of HCC cells (***Figure 3C***), which was also validated by the CCK-8 assay (***Figure 3D-E***). Moreover, EdU assay revealed that the knockdown of AC099850.3 reduced the level of EdU-positive cells, which suggests that it inhibits cell proliferation (***Figure 3F***). Also, flow cytometry results revealed that apoptosis rate in the si-AC099850.3 group increased significantly when compared with that of the control group (***Figure 3G***). Moreover, the results of *in vivo* experiments revealed that the mice implanted with si-AC099850.3 cells tended to have smaller tumors (***Figure 3H***). HE staining showed that the tumor with si-AC099850.3 had a lower section area than the control group (***Figure 3I***). In parallel, Ki67 staining revealed that the silencing of AC099850.3 reduced Ki67 positive cells in xenograft tumors (***Figure 3J-K***).

### Knockdown of AC099850.3 suppresses the invasion and migration of HCC cells

**Figure 4:**
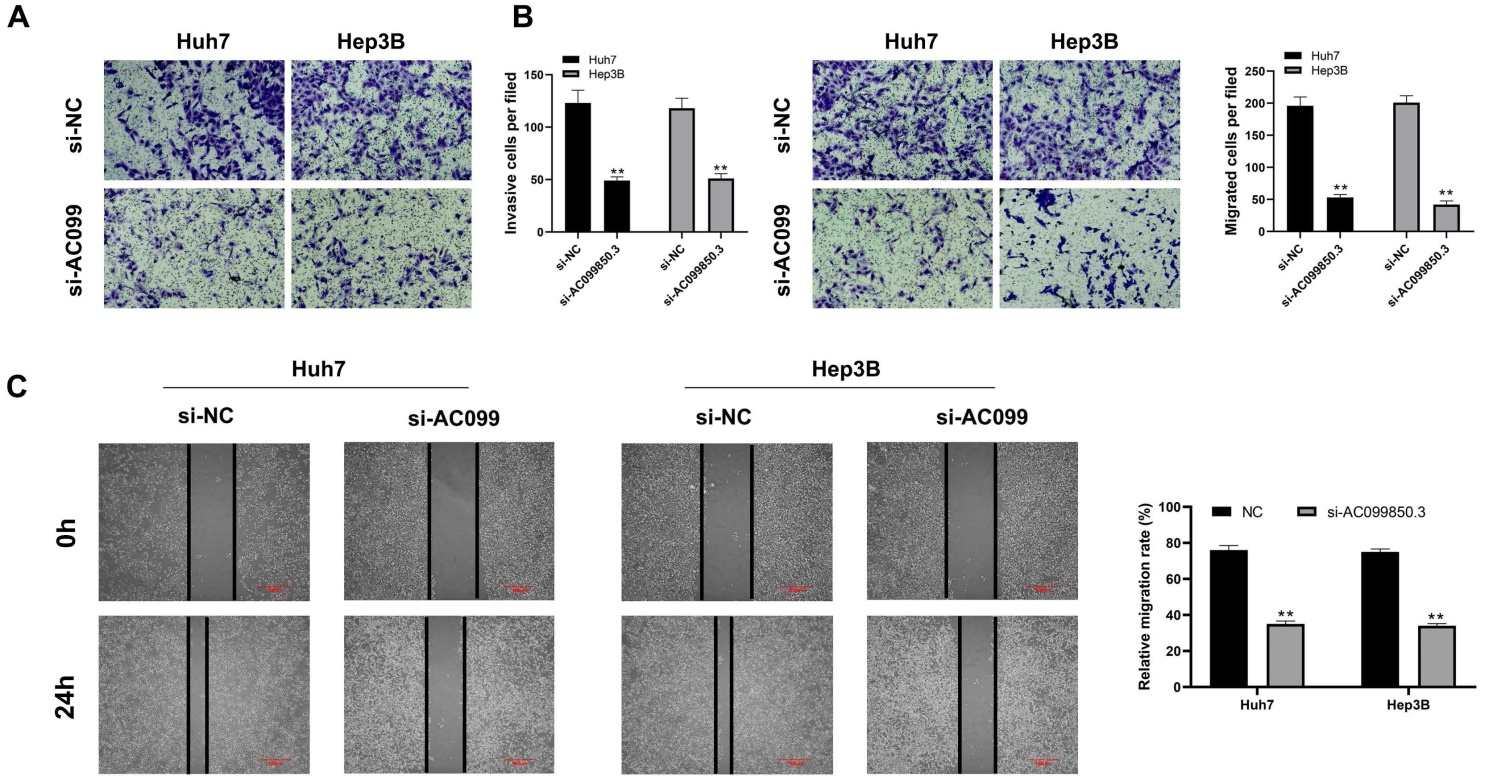
AC099850.3 facilitate cell metastasis potential in HCC. **Notes: A-B:** Downregulation of AC099850.3 reduced the number of invasion and migration cells in the transwell assay;** C:** Wound-healing assay performed between NC and si-AC099850.3 group. **Notes:** All experiments were repeated at least three times

The correlation between AC099850.3 and cell metastasis was determined. Transwell assay revealed that knockdown of AC099850.3 decreased the number of invasive and migratory cells significantly (***Figure 4A-B***). Similarly, the wound healing assay revealed that wound healing area in the AC099850.3 knockdown group was smaller than that of the control group after 24 hours (***Figure 4C***).

### AC099850.3 activates PI3K/AKT signaling pathway

**Figure 5:**
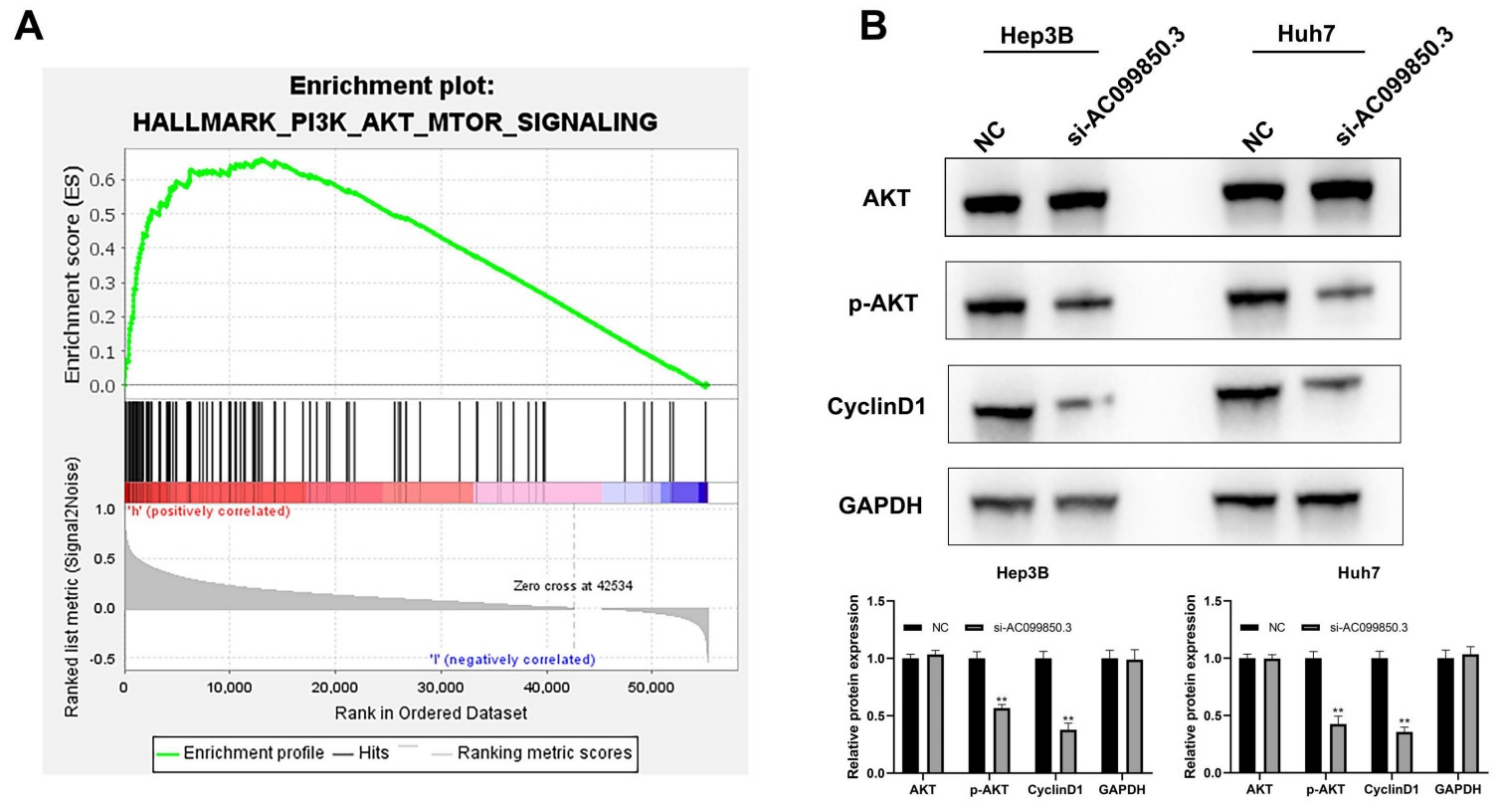
AC099850.3 affect the activity of the PI3K/AKT pathway. **Notes: A:** GSEA analysis between high and low AC099850.3 group;** B:** Knockdown of AC099850.3 inhibit the activity of PI3K/AKT pathway. **Notes:** All experiments were repeated at least three times

The results of GSEA revealed that the PI3K/AKT signaling pathway was associated with the biological function of AC099850.3 (***Figure 5A***). Consequently, the key molecules involved in the PI3K/AKT signaling pathway were analyzed by western blotting (AKT, p-AKT, and cyclin D1). The results revealed that the knockdown of AC0998503 decreased the protein levels of p-AKT and cyclin D1, implying that AC099850.3 could activate the AKT signaling pathway (***Figure 5B***). The results suggest that AC099850.3 promotes malignant biological behavior of HCC cells by regulating the AKT signaling pathway.

### *PRR11* is a target gene of AC099850.3

**Figure 6:**
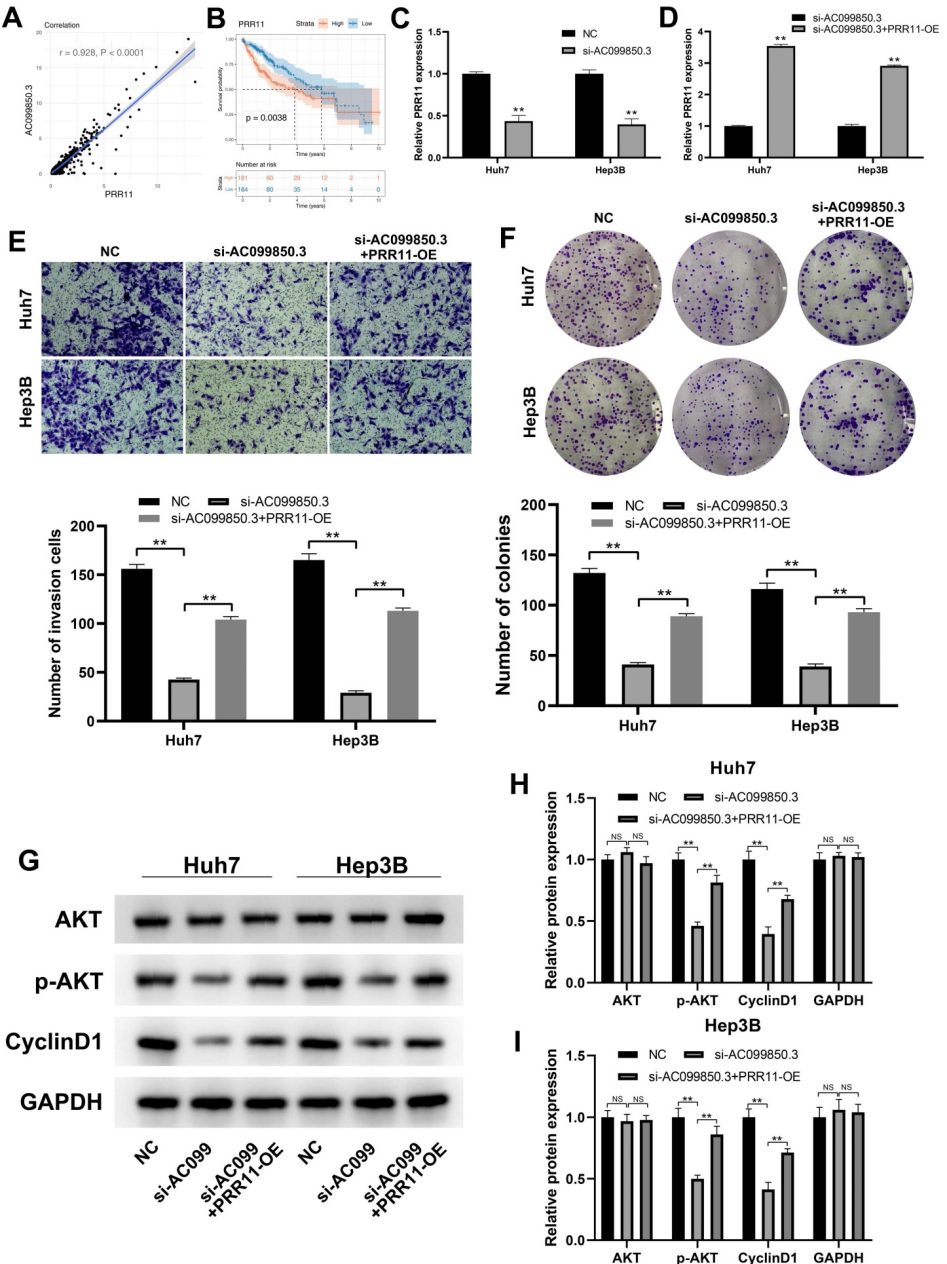
PRR11 is a downstream gene of AC099850.3. **Notes: A:** PRR11 is significantly positive with AC099850.3;** B:** Kaplan-Meier survival curves showed that patients with high IGGL2-AS1 tend to have a worse prognosis;** C:** PRR11 expression level in NC and si-AC099850.3 cells;** D:** PRR11 expression level in si-AC099850.3 and si-AC099850.3+PRR11-OE cells; **E:** Transwell assay was performed in the NC, si-AC099850.3 and si-AC099850.3+PRR11-OE cells;** F:** Colony formation assay was performed in the NC, si-AC099850.3 and si-AC099850.3+PRR11-OE cells; **G:** The protein level of the key biomarker in PI3K/AKT pathway in NC, si-AC099850.3 and si-AC099850.3+PRR11-OE cells. **Notes:** All experiments were repeated at least three times

PRR11 exhibited a strong positive correlation with AC099850.3 based on the analysis of transcription profiling data obtained from the TCGA-LIHC database (***Figure 6A***, r = 0.928, P < 0.0001). PRR11 has been reported to promote the progression of liver cancer, and Kaplan-Meier survival curve analysis revealed that the PRR11 was associated with a shorter survival time (***Figure 6B***, P < 0.01). Therefore, we hypothesized that the oncogenic effect of AC099850.3 could be partially associated with PRR11. Subsequently, we detected PRR11 expression levels in control and si-AC099850.3 cells. The results revealed a considerable decrease in PRR11 mRNA expression levels in the AC099850.3 knockdown group (***Figure 6C***). The expression of PRR11 in AC099850.3-knockdown cells was restored and the overexpressed efficiency was validated using qRT-PCR assays (***Figure 6D***). Transwell assay revealed that the overexpression of PRR11 antagonized the anticancer effect caused by down-regulation of AC099850.3 (***Figure 6E***). A similar trend was also observed in the colony formation assay (***Figure 6F***). In addition, previous studies revealed that PRR11 could promote activation of the PI3K/AKT pathway 14. Variations in the expression of PI3K/AKT marker in AC099850.3-knockdown cells with PRR11 re-expression were analyzed considering the PI3K/AKT pathway was the downstream effector of AC099850.3. Western blotting assay revealed that the protein levels of p-AKT and cyclin D1 were up-regulated in AC099850.3-knockdown cells after restoration of PRR11 expression (***Figure 6G-I***). Overall, the results of the current study revealed that AC099850.3 exerted oncogenic effects in liver cancer cells through the PRR11/PI3K/AKT pathway.

### AC099850.3 act as an immune-related gene in HCC

**Figure 7:**
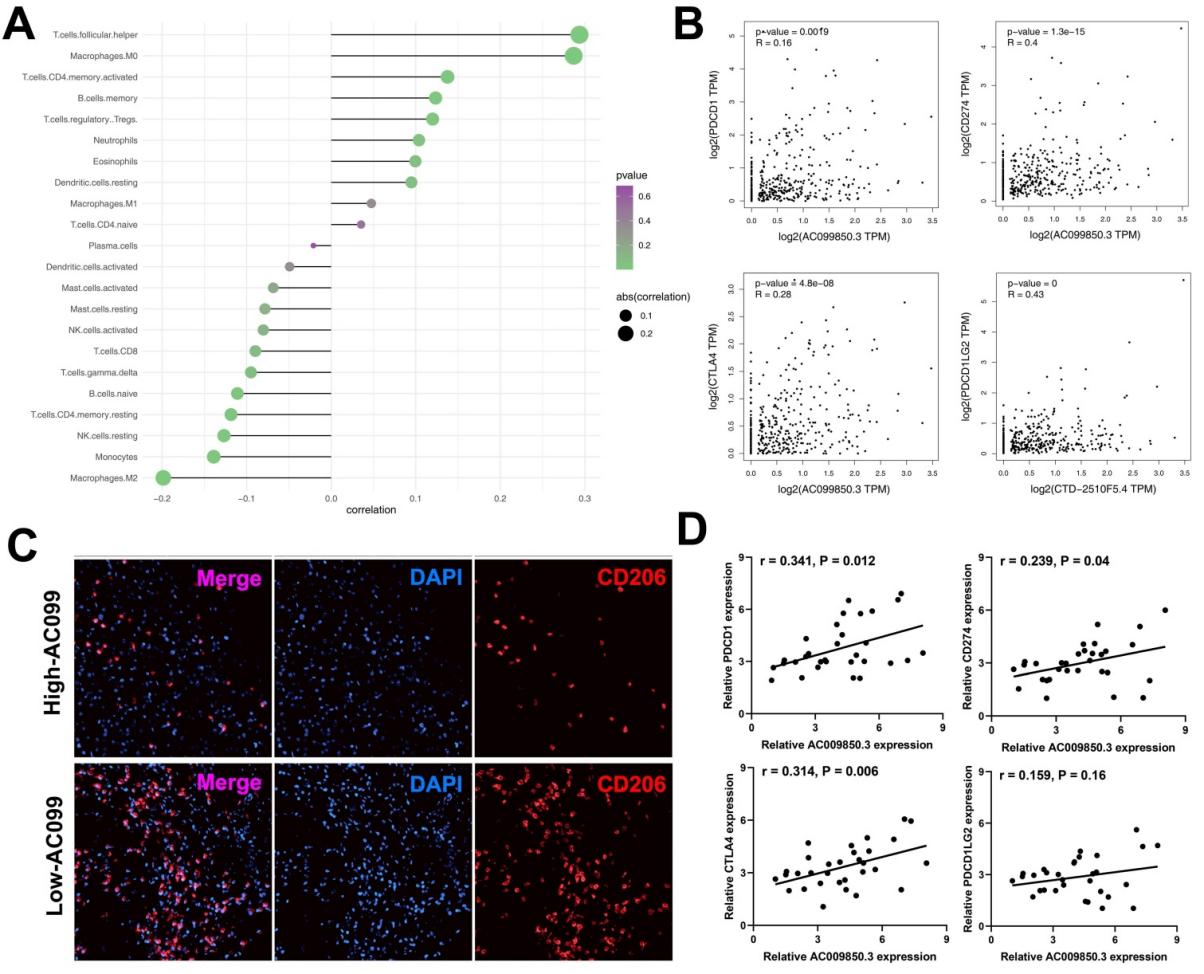
Immune relevance analysis of AC099850.3 in HCC. **Notes: A:** Immune infiltration analysis of AC099850.3 to explore its effect on tumor immune microenvironment; **B:** Immune checkpoint analysis of AC099850.3; **C:** M2 macrophages content in high and low AC099850.3 samples; **D:** qRT-PCR was performed to evaluate the correlation between AC099850.3 and four immune checkpoint molecules. **Notes:** All experiments were repeated at least three times

The interaction between the immune microenvironment and tumor cells influences tumor development. Therefore, we investigated the underlying association between AC099850.3 and tumor immune microenvironment. Immune infiltration analyses results revealed that AC099850.3 was positively correlated with T follicular helper cells, M0 macrophages, CD4^+^ memory T cells, memory B cells but negatively correlated with M2 macrophages, monocytes, natural killer (NK) cells, and CD8^+^ T cells (***Figure 7A***). Immune checkpoint therapy has been a promising field of cancer treatment. In addition, we determined the correlation between AC099850.3 and immune checkpoints. Strikingly, a significantly positive correlation was observed between AC099850.3 and four major checkpoint proteins, PD-L1 (R = 0.16, P < 0.01), PD-1 (R = 0.4, P < 0.01), CTLA4 (R = 0.28, P < 0.01), and PD-L2 (R = 0.28, P < 0.01) (***Figure 7B***). M2 macrophages exhibited the highest negative correlation with AC099850.3 and were therefore selected for further validation. Immunofluorescence results revealed that the tissues with high expression of AC099850.3 tended to have relatively low M2 macrophage infiltration levels (***Figure 7C***). Furthermore, we validated the correlation between AC099850.3 through PCR assay. The result showed that PD-1, PD-L1, CTLA4, and PD-L2 might positively correlate with AC099850.3 based on 31 liver cancer tissue (***Figure 7D***, PDCD1: r = 0.341, P = 0.012; CD274: r = 0.239, P = 0.04; CTLA4: r = 0.314, P = 0.006; PDCD1LG2: r = 0.159, P = 0.16).

## Discussion

Liver cancer is a malignancy with high lethality globally and the most prevalent pathologic subtype of HCC 15. The incidence of obesity, type 2 diabetes, nonalcoholic fatty, and other related liver diseases has been increasing steadily due to lifestyle changes, leading to an increased burden of liver cancer 16. Consequently, liver cancer has been a critical public health problem worldwide; therefore, it is crucial to identify novel diagnostic biomarkers and therapeutic targets of liver cancer.

To the best of our knowledge, this is the first study to investigate the biological role of the lncRNA AC099850.3 in HCC. In the present study, lncRNA AC099850.3 was first identified as the candidate gene. Further analysis revealed that AC098850.3 was correlated with poor prognosis and clinical features. A relatively high expression level of AC099850.3 was observed in HCC tissues and cell lines. *In vitro* experiments revealed that the knockdown of AC099850.3 significantly impaired the proliferation and invasion ability of HCC cells. The knockdown of AC099850.3 inhibited HCC growth *in vivo*. Furthermore, flow cytometry revealed that a low expression level of AC099850.3 could enhance cell apoptosis.

Numerous previous studies have reported the role of AC099850.3 in cancer. For example, Zhou and colleagues revealed that the lncRNA AC099850.3 was associated with the prognosis of tongue squamous cell carcinoma and the mechanism of competing endogenous RNAs 17. Jiang and colleagues revealed that AC099850.3 was correlated with cell autophagy and could predict the overall survival of patients with oral and oropharyngeal squamous cell carcinoma 18. Additionally, Jia and colleagues revealed the prognostic association of AC099850.3 in HCC through bioinformatics analysis 19. The present study initially investigated the biological function of AC099850.3 in HCC by performing a series of cell phenotyping experiments. The lncRNA AC099850.3 can promote the malignant behavior of HCC cells, in turn, serving as a potential therapeutic target.

The PI3K/AKT signaling pathway has been reported to perform essential functions in cancer progression. For instance, Blattner et al. revealed that speckle-type POZ protein mutation could facilitate tumor metastasis by modulating PI3K/AKT and AR signaling 20. Narayanankutty comprehensively reviewed preclinical and clinical evidence, and concluded that the PI3K/AKT pathway was a potential therapeutic target for colorectal cancer 21. Moreover, drugs targeting PI3K/AKT pathway, including pan-PI3K inhibitors and isoform-specific inhibitors, have achieved satisfactory tumor treatment efficacy 22. Ippen and colleagues assessed the efficacy of GDC-0084, a dual PI3K/AKT pathway inhibitor in breast cancer using in vitro and in vivo models, and the results revealed that GDC-0084 was a potential treatment option, especially for patients with brain metastases 23. Also, Tarantelli et al. revealed that the novel dual PI3K/AKT inhibitor, PQR309 exhibited preclinical antitumor activity in lymphomas, and could be developed further for lymphoma therapy 24. Our results revealed that AC099850.3 could partially act as an oncogene depending on the PI3K/AKT pathway. Considering the critical role of the PI3K/AKT pathway in cancer, the results of the current study suggest that AC099850.3 could be a potential therapeutic target for liver cancer.

Analyses of immune cells revealed a negative correlation between AC099850.3 and NK cells and CD8^+^ T cells, which could be attributed to the cancer-promoting effect of AC099850.3. NK cells are abundant in healthy liver tissues and play a crucial role in the surveillance of HCC 25. NK cells have been reported to exhibit considerable cytotoxic effects associated with cell apoptosis and patient survival 26, 27. Generally, a high abundance of CD8^+^ T cells in tumor tissues results in a better prognosis of patients 28. Previous studies have demonstrated that the inhibition of CD8^+^ T cells attenuates tumor-specific immune response, thereby affecting proliferation and metastasis 29. In addition, a positive correlation was observed between AC099850.3 and the main immune checkpoint molecules (CTLA4, PD-1, PD-L1, and PD-L2). Immune checkpoint inhibitors are a novel and promising targets of tumor therapy 30. The lncRNA AC099850.3 could be a potential novel immune-therapeutic target for HCC due to its significant association with HCC tumor immune microenvironment.

## Conclusion

The present study revealed that the lncRNA AC099850.3 was up-regulated in HCC tissues and its high expression was associated with poor prognosis of patients with HCC. The lncRNA AC099850.3 significantly increased the proliferation and invasion ability of HCC cells through the PRR11/PI3K/AKT pathway. Moreover, lncRNA AC099850.3 influenced the abundance of various immune cells in the tumor microenvironment, especially M2 macrophage infiltration and was positively correlated with immune checkpoint molecules (PD-1, PD-L1. PD-L2, and CTLA4). The lncRNA AC099850.3 could be a potential therapeutic target for HCC.

## Figures and Tables

**Table 1:** The clinic parameters of enrolled patients obtained from TCGA-LIHC

Clinical parameters		Number of patients (n)	Proportion of patients (%)
Age	<=60	180	47.7
	>60	196	51.9
	Unknow	1	0.3
Gender	Female	122	32.4
	Male	255	67.6
Grade	G1	55	14.6
	G2	180	47.7
	G3	124	32.9
	G4	13	3.4
	Unknow	5	1.3
Stage	Stage I	175	46.4
	Stage II	87	23.1
	Stage III	86	22.8
	Stage IV	5	1.3
	Unknow	24	6.4
T-stage	T1	185	49.1
	T2	95	25.2
	T3	81	21.5
	T4	13	3.4
	Unknow	3	0.8
M-stage	M0	272	72.1
	M1	4	1.1
	Unknow	101	26.8
N-stage	N0	257	68.7
	N1	4	1.1
	Unknow	116	30.8

## Abbreviations

HCC: hepatocellular carcinoma
TCGA: The Cancer Genome Atlas
GSEA: gene set enrichment analysis
LIHC: liver hepatocellular carcinoma
LncRNA: long non-coding RNA
